# Andes hantavirus aboard an expedition cruise ship: A sentinel event for travel medicine and maritime biosurveillance

**DOI:** 10.17179/excli2026-9613

**Published:** 2026-07-30

**Authors:** Paulo Ricardo Martins-Filho, Thialla Andrade Carvalho, Adriano Antunes de Souza Araújo, Lucindo José Quintans-Júnior

**Affiliations:** 1 Investigative Pathology Laboratory, Federal University of Sergipe, Lagarto, SE, Brazil; 2 Laboratory of Pharmaceutical Assays and Toxicity, Federal University of Sergipe, São Cristóvão, SE, Brazil; 3 Laboratory of Neuroscience and Pharmacological Assays, Federal University of Sergipe, São Cristóvão, SE, Brazil

## ⁯⁯

The recent outbreak of Andes hantavirus linked to the expedition cruise ship MV *Hondius*, during an itinerary spanning Argentina, Antarctica, and remote South Atlantic islands, represents the first documented cluster of hantavirus infection aboard a cruise vessel and constitutes an important event in the management of emerging infectious diseases in international travel settings. Unlike traditional cruise-associated outbreaks, which typically involve gastrointestinal or respiratory pathogens, this event involved a rare zoonotic agent with limited but recognized potential for person-to-person transmission. By June 2026, the outbreak had been linked to 13 confirmed cases and three deaths, prompting large-scale international contact tracing, passenger repatriation, and prolonged monitoring of exposed travelers across multiple countries. The response included quarantine measures and coordinated follow-up by national and international public health authorities, reflecting the complexity of managing rare infectious threats in highly mobile populations.

Hantaviruses are primarily acquired through exposure to aerosolized rodent excreta, but Andes virus (ANDV), endemic to Argentina and Chile, differs from other hantaviruses because of its documented, though uncommon, capacity for interpersonal transmission. Virological characterization of the ARG-Epuyén strain associated with the 2018-2019 Argentine outbreak demonstrated high infectiousness and efficient mammalian transmission, suggesting that certain strains may have greater epidemic potential under specific conditions (Coelho et al., 2025[1]). Prospective clinical evidence has also identified systemic viremia and infectious viral particles in saliva, gingival crevicular fluid, nasopharyngeal secretions, and urine, supporting plausible mechanisms for close-contact transmission in confined environments (Ferrés et al., 2024[3]).

Nevertheless, epidemiological evidence remains limited. A systematic review concluded that evidence for human-to-human transmission is weak, restricted to ANDV, and geographically confined to selected regions of Argentina and Chile (Toledo et al., 2022[4]). In this context, the significance of the MV *Hondius* outbreak lies in demonstrating that even limited and geographically constrained transmission potential may become operationally relevant when ANDV is introduced into expeditionary maritime settings characterized by prolonged close contact, ecological exposure, and complex international passenger movement.

This event also reflects broader ecological determinants of hantavirus emergence in Latin America. Climatic variability, rainfall, environmental disruption, and rodent reservoir dynamics appear more relevant to transmission risk than biodiversity alone (Vadell et al., 2020[5]; Douglas et al., 2021[2]). Expeditionary routes through environmentally dynamic and remote regions may therefore create unique interfaces between travelers and geographically specific zoonotic hazards. The MV *Hondius* itinerary highlights the growing interaction between frontier tourism and ecological settings where zoonotic pathogens may remain underrecognized by conventional infectious disease surveillance strategies.

This outbreak exposes potential structural limitations in maritime infectious disease preparedness. Existing cruise health protocols have largely evolved around norovirus, influenza, and, more recently, SARS-CoV-2, with comparatively limited emphasis on geographically specific zoonotic threats. Pre-embarkation screening rarely incorporates ecological exposure history, rodent-associated risk, or destination-specific zoonoses, particularly for expeditionary routes involving remote endemic regions.

The outbreak also exposed challenges in the post-travel management of exposed individuals. Differences in quarantine and monitoring strategies across jurisdictions highlight the lack of harmonized approaches for rare pathogens with limited but plausible person-to-person transmission. The MV *Hondius* outbreak demonstrates how a zoonotic infection acquired during expedition travel can rapidly evolve into a multinational public health event requiring coordinated surveillance, traveler follow-up, quarantine measures, and cross-border response.

## Declaration

### Author contributions

P.R.M.F. contributed to conceptualization, writing - original draft, and writing - review & editing. T.A.C. contributed to writing - original draft and writing - review & editing. A.A.S.A. contributed to writing - review & editing and supervision. L.J.Q.Jr contributed to writing - review & editing and supervision.

### Conflict of interest

We declare no competing interests.

### Funding

There is no funding source.

### Acknowledgments

P.R.M.F., L.J.Q.Jr, and A.A.S.A. are research productivity fellows at the Conselho Nacional de Desenvolvimento Científico e Tecnológico (CNPq), Brazil.

### Artificial Intelligence (AI) - assisted technology

The authors used OpenAI's ChatGPT to assist with readability improvements, grammar correction, and style editing.
